# Neighborhood environmental factors linked to hospitalizations of older people for viral lower respiratory tract infections in Spain: a case-crossover study

**DOI:** 10.1186/s12940-022-00928-x

**Published:** 2022-11-08

**Authors:** Alejandro Álvaro-Meca, Daniel Sepúlveda-Crespo, Rosa Resino, Pablo Ryan, Isidoro Martínez, Salvador Resino

**Affiliations:** 1 Departamento de Medicina Preventiva y Salud Pública, Facultad de Ciencias de la Salud, Universidad Rey Juan Carlos, Alcorcón, Madrid, Spain; 2 Centro de Investigación Biomédica en Red de Enfermedades Infecciosas (CIBERINFEC), Instituto de Salud Carlos III, Madrid, Spain; 3 Unidad de Infección Viral e Inmunidad, Centro Nacional de Microbiología, Instituto de Salud Carlos III, Majadahonda, Madrid, Spain; 4 Departamento de Geografía, Facultad de Geografía e Historia, Universidad Complutense de Madrid, Madrid, Spain; 5 Hospital Universitario Infanta Leonor, Madrid, Spain; 6 Universidad Complutense de Madrid (UCM), Madrid, Spain; 7 Instituto de Investigaciones Sanitarias Gregorio Marañón (IiSGM), Madrid, Spain

**Keywords:** ICD-9-CM, Older people, Air pollution, Temperature, Respiratory virus, Lower respiratory tract infection

## Abstract

**Background:**

Lower respiratory tract viral infection (LRTI) is a significant cause of morbidity-mortality in older people worldwide. We analyzed the association between short-term exposure to environmental factors (climatic factors and outdoor air pollution) and hospital admissions with a viral LRTI diagnosis in older adults.

**Methods:**

We conducted a bidirectional case-crossover study in 6367 patients over 65 years of age with viral LRTI and residential zip code in the Spanish Minimum Basic Data Set. Spain’s State Meteorological Agency was the source of environmental data. Associations were assessed using conditional logistic regression. *P*-values were corrected for false discovery rate (*q*-values).

**Results:**

Almost all were hospital emergency admissions (98.13%), 18.64% were admitted to the intensive care unit (ICU), and 7.44% died. The most frequent clinical discharge diagnosis was influenza (90.25%). LRTI hospital admissions were more frequent when there were lower values of temperature and O_3_ and higher values of relative humidity and NO_2_. The regression analysis adjusted by temperatures and relative humidity showed higher concentrations at the hospital admission for NO_2_ [compared to the lag time of 1-week (*q*-value< 0.001) and 2-weeks (*q*-value< 0.001)] and O_3_ [compared to the lag time of 3-days (*q*-value< 0.001), 1-week (*q*-value< 0.001), and 2-weeks (*q*-value< 0.001)] were related to a higher odds of hospital admissions due to viral LRTI. Moreover, higher concentrations of PM_10_ at the lag time of 1-week (*q*-value = 0.023) and 2-weeks (*q*-value = 0.002), and CO at the lag time of 3-days (*q*-value = 0.023), 1-week (*q*-value< 0.001) and 2-weeks (*q*-value< 0.001)], compared to the day of hospitalization, were related to a higher chances of hospital admissions with viral LRTI.

**Conclusion:**

Unfavorable environmental factors (low temperatures, high relative humidity, and high concentrations of NO_2_, O_3_, PM_10_, and CO) increased the odds of hospital admissions with viral LRTI among older people, indicating they are potentially vulnerable to these environmental factors.

**Graphical Abstract:**

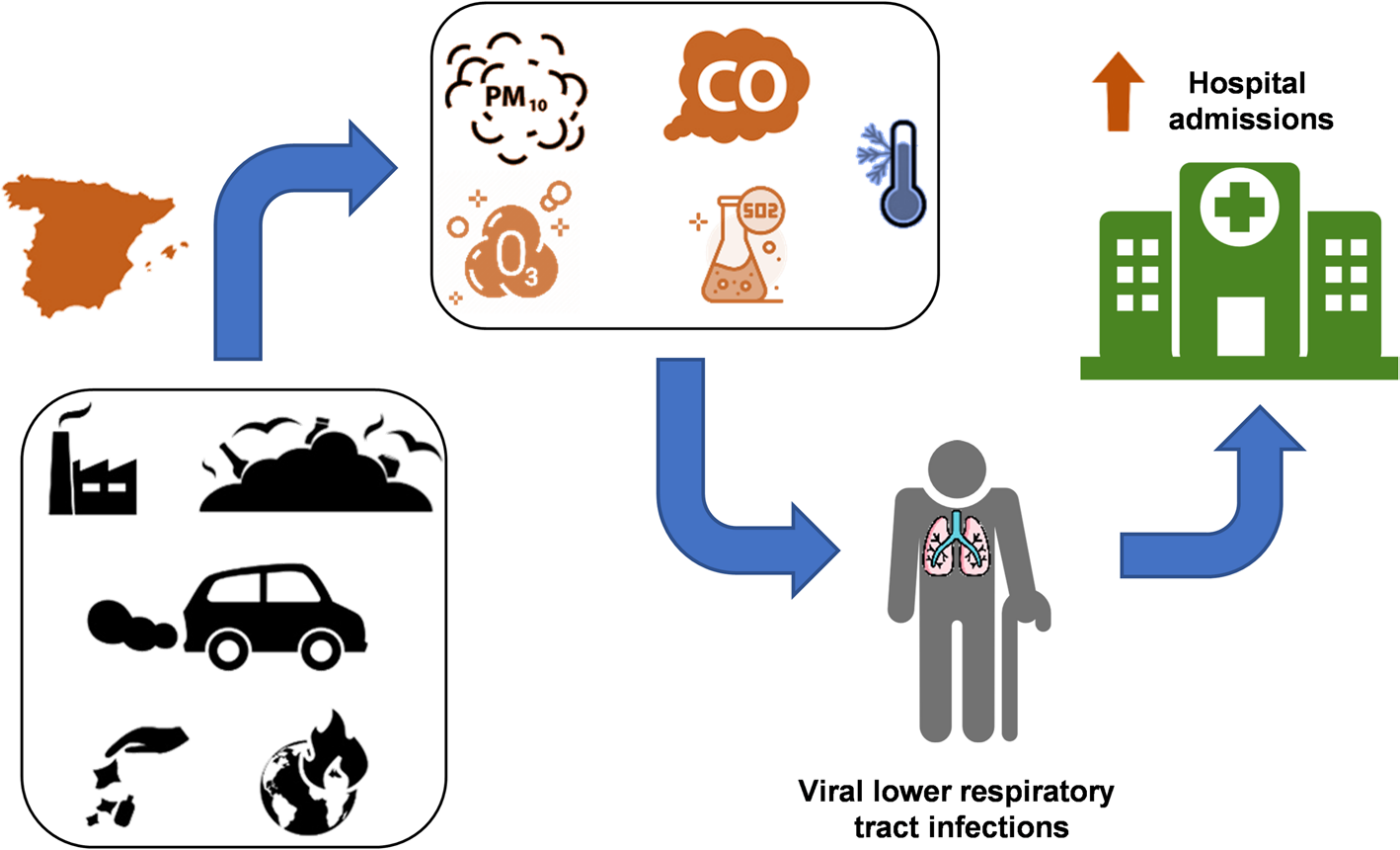

**Supplementary Information:**

The online version contains supplementary material available at 10.1186/s12940-022-00928-x.

## Introduction

Lower respiratory tract infections (LRTIs) are a significant cause of hospitalizations, morbidity, and mortality worldwide, particularly in older adults over 65 years old [1]. Older people are at increased risk due to a less efficient immune system (immunosenescence), a progressive decrease in respiratory function, a greater predisposition to respiratory infections, and being more prone to develop complications [2, 3]. LRTI includes acute bronchiolitis, bronchitis, and pneumonia, causing severe respiratory failure and leading to hospitalization and possible death [4].

The incidence of bacterial LRTIs in older people has substantially decreased in the last decades due to vaccination programs and antibiotic treatment. Thus, the most frequent etiological agents responsible for LRTIs are respiratory viruses, such as the respiratory syncytial virus (RSV) and influenza virus (Flu), among others [1, 4]. Viral LRTI leads to an uncontrolled host immune response, damaging the lung epithelium and decreasing respiratory gas exchange [5].

Knowing all the risk factors for viral LRTIs can help prevent and improve their management in the healthcare system. Environmental factors are crucial in developing lung diseases, including respiratory infections [2, 6, 7]. The air pollutants for public health concerns are carbon monoxide (CO), nitrogen dioxide (NO_2_), ozone (O_3_), particulate matter up to 10 μm in size (PM_10_), and sulfur dioxide (SO_2_), among others [2, 6]. Several studies have shown associations between short-term exposure to outdoor air pollutants and hospitalizations, emergency room visits, or home health visits for respiratory morbidity (pneumonia, chronic obstructive pulmonary disease, asthma) in people aged 65 years and older [8].

Climatic factors are other contributors to human morbidity and mortality from lung disease. Viral LRTIs have a seasonal pattern in older people [9, 10]. Seasonal environmental changes (temperature, wind speed, humidity, thunderstorms) are associated with a higher probability of infections [7]. Seasonal temperature fluctuations and relative humidity, particularly in winter, affect the transmission of respiratory viruses and host susceptibility [11].

Several studies have shown that climatic changes can affect air quality and significantly impact the concentration of air pollutants, their mixture, and how they are dispersed or deposited [12]. The interaction of air pollution and climate factors affects pathogens’ survival time, increasing the host’s susceptibility [13, 14]. This relationship between environmental factors (climatic factors and outdoor air pollution) and viral LRTIs in the older population may not be uniform, requiring multivariate analysis considering the maximum number of climatic and environmental factors.

Scientific publications on environmental pollution and respiratory diseases are abundant. Air pollutant exposure may alter respiratory system responses against viral infection by modulating appropriate host defense responses and increasing viral LRTI and associated morbidity [15]. However, information about the older population with viral LRTI is scarce [1]. This study aimed to analyze the association between short-term exposure to environmental factors and hospital admissions with viral LRTI diagnosis in older people aged 65 years or older before the Coronavirus Disease 2019 (COVID-19) era.

## Material and methods

### Study design

We conducted a bidirectional case-crossover study (all patients serve as their controls) in individuals aged 65 years or older who had a hospital admission due to viral LRTI in Spain during 2013–2015. This study was approved by the Research Ethics Committee (Comité de Ética de la Investigación; CEI PI 81_2021) of the Instituto de Salud Carlos III (Madrid, Spain). All the extracted information was completely anonymous and did not require the patients’ consent.

### Epidemiological and clinical data

Clinical information was obtained from the Spanish Minimum Basic Data Set (MBDS) facilitated by the Health Information Institute of the Spanish Ministry of Health, Consumer Affairs and Social Welfare (MHCSW), previously described [16]. This anonymized database provides discharge diagnoses, procedures, and epidemiological information. Diagnoses are made following the standardized methods of each hospital. Data were recorded using the *International Classification of Diseases, 9th ed, Clinical Modification* (ICD-9-CM). One of the fields that the MBDS provides is the residential zip code. However, in some registries, this field was incorrectly collected or absent in 30% of the patients, so it was impossible to geolocate them.

### ICD-9-CM codes of the outcome variable

We selected older patients who had a primary diagnosis of viral LRTI [Flu (487.0, 487.1, 488.01, 488.02, 488.11, 488.12, 488.81, and 488.82), RSV (079.6, 466.11, and 480.1), viral pneumonia (480.0, 480.1, 480.2, 480.8, and 480.9), and acute bronchiolitis (466.11 and 466.19)] or a primary diagnosis of acute respiratory failure (518.81) with a secondary diagnosis of viral LRTI (Supplementary Table 1).

The outcome variable was hospital admission with viral LRTI. Patients were never hospitalized in the three control times before hospital admission (3 days, 1-week, and 2-weeks).

### Environmental data

Environmental information was collected from the Spanish State Meteorological Agency (AEMET; http://www.aemet.es/en/), which provides data from 880 meteorological stations throughout the Spanish territory [17] as geolocation, climatic data, and outdoor air pollutants. The quality of the AEMET data meets the criteria of the European Environment Agency [18]. The environmental data for each patient were obtained from the weather station closest to the patient’s residential zip code.

The link in space-time between environmental factors and MBDS data was established as follows: i) The environmental data [temperature (°C) and relative humidity (%)] and ambient air pollutants [SO_2_ (μg/m^3^), CO (μg/m^3^), NO_2_ (μg/m^3^), O_3_ (μg/m^3^), PM_10_ (μg/m^3^)] from the meteorological stations distributed throughout the territory were geolocated in space as a reference point (latitude-longitude). ii) Each patient in the study had their spatial location through the residential zip code (geographical area), from which the centroid was extracted, and geolocation in space as a reference point (latitude-longitude) was obtained. iii) Once both data sources were geolocated in space, each patient was linked to the meteorological station closest to their home. iv) The MBDS had the date of hospital admission and each meteorological station of the measurement date, so the link of dates was simple. The mean distance from each residential zip code to its nearest meteorological station was 8.99 km 95% CI (8.69, 9.28).”

### Statistical analysis

In this bidirectional case-crossover analysis, three short control times (3 days, 1-week, and 2-weeks before hospital admission), compared to the day of hospital admission, were used to estimate the risk of hospital admission with viral LRTI related to acute exposure to environmental factors [19]. A symmetric bidirectional sampling with control periods before and after hospital admission was used to adjust the long-term trend and seasonality impact on variable exposures [20, 21]. Since the time between infection, symptoms, and hospital admission is unknown in our study and may vary over a wide range, these three control times were analyzed separately to cover the wide range of days at risk. In each of these three control times, we calculated the mean value of each environmental factor in a bidirectional way taking into account three days (one day before, the day of the study, and one day after) to mitigate extreme levels. With this design, it was unnecessary to include invariant confounding factors because each individual is their control [20, 21].

The conditional logistic regression (CLR) was used to evaluate the association between environmental factors (climatic factors and ambient air pollutants) and hospital admissions with viral LRTI by univariate analysis (each environmental factor separately) and multivariate analysis (adjusted by temperature and relative humidity). This test provides the measure of effect, called the odds ratio (OR), and its 95% confidence interval (95%CI), calculated using the exact method by dividing the exposure level at the hospital admission by the exposure level at the control time, implying only individuals with variations in variable exposures provide information. Thus, an OR > 1 indicated higher odds when the environmental variable was augmented at the hospital admission or reduced at the control lag time, and an OR < 1 showed higher odds when the environmental feature was increased at the control lag time or decreased at the hospital admission. Many OR values tended to 1, making interpretation difficult, which is why relative humidity (%)], SO_2_ (μg/m^3^), CO (μg/m^3^), NO_2_ (μg/m^3^), O_3_ (μg/m^3^), and PM_10_ (μg/m^3^) were log_2_-transformed. The assumption of linearity was confirmed by plotting the Martingale residuals on the Y axis against continuous covariates on the X axis.

Statistical analysis was performed using the R statistical software v3.5.2 [22], with the *clogit* function from a package for survival analysis (version 3.4–0, https://CRAN.R-project.org/package=survival) [23]. All tests were two-tailed, and *p*-values were corrected by the Benjamini and Hochberg procedure’s false discovery rate (*q*-values).

## Results

### Population characteristics

We found 6367 hospital admissions of patients over 65 with viral LRTI and residential zip code. The patients’ median was 78 years old, and half were men (50.51%). The Charlson comorbidity index was 2.14. The two most frequent comorbidities were congestive heart failure (19.63%) and diabetes (32.73%). Almost all were emergency hospital admissions (98.13%), 18.64% were admitted to the intensive care unit (ICU), and 7.44% died. The most frequent clinical discharge diagnosis was Flu (90.25%) (Table 1).

**Table 1:** Summary of the epidemiological and clinical characteristics of patients with hospital admission due to lower respiratory tract viral infections in Spain (2013–2015)

Description	Data
No.	6367
Gender (male)	3216 (50.51)
Age (years)	78.37 (78.18; 78.56)
Alcohol intake	1372 (21.55)
Smoker	495 (7.77)
Comorbidities	
Charlson index	2.14 (2.09; 2.18)
Myocardial infarction	234 (3.68)
Congestive heart failure	1250 (19.63)
Peripheral vascular disease	347 (5.45)
Cerebrovascular disease	393 (6.17)
Dementia	287 (4.51)
Chronic pulmonary disease	2184 (34.3)
Rheumatic disease	207 (3.25)
Peptic ulcer disease	33 (0.52)
Mild liver disease	242 (3.8)
Moderate or severe liver disease	34 (0.53)
Diabetes without chronic complication	1856 (29.15)
Diabetes with chronic complication	228 (3.58)
Hemiplegia or paraplegia	19 (0.3)
Renal disease	1121 (17.61)
Any malignancy, including lymphoma and leukemia, except malignant neoplasm of skin	597 (9.38)
Metastatic solid tumor	104 (1.63)
AIDS/HIV	10 (0.16)
Clinical events during admission	
Emergency admission	6248 (98.13)
Surgical condition	75 (1.18)
ICU admission	1187 (18.64)
Length of stay (days)	9.16 (8.94; 9.39)
In-hospital death	474 (7.44)
Clinical discharge diagnosis	
Acute lower respiratory infection	6313 (99.15)
Respiratory syncytial virus	134 (2.1)
Influenza	5746 (90.25)
Viral pneumonia	380 (5.97)
Acute bronchiolitis	287 (4.51)
Acute respiratory failure	2411 (37.87)

Values are expressed as absolute number (percentage) and median (P25th; P75th)

### Environmental features and viral LRTI hospital admissions

**Fig. 1:**
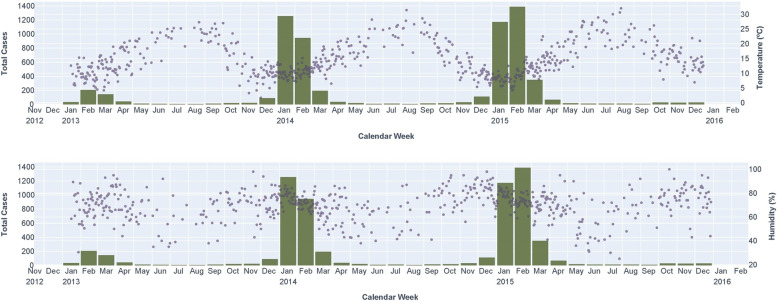
Summary of LRTI hospital admissions by months (green bars) and levels of climatic factors (temperature and relative humidity) by day (gray dots) in older people > 65 years from 2013 to 2015

LRTI hospital admissions were more frequent with low temperatures and high relative humidity (Fig. 1). In the univariate regression analysis (Table 2), the temperature showed significant values of OR > 1, indicating lower values at the lag time of 3-days (*q*-value = 0.014), 1-week (*q*-value< 0.001), and 2-weeks (*q*-value< 0.001) than at the day of hospitalization were related to a higher odds of hospital admissions due to viral LRTI. Moreover, the relative humidity showed a significant value of OR < 1, indicating higher values of relative humidity at the lag time of 2-weeks (*q*-value< 0.001) before the day of the hospitalization increased the odds of LRTI hospital admission.

**Table 2:** Summary of univariate associations between climatic factors and hospital admissions for lower respiratory tract viral infections in Spain (2013–2015)

	OR (95% CI)	*p*-value	*q*-value
3 days			
Log_2_ temperature (°C)	1.02 (1.01; 1.04)	**0.008**	**0.014**
Log_2_ humidity (%)	1.00 (0.99; 1.01)	0.670	0.704
1 week			
Log_2_ temperature (°C)	1.04 (1.03; 1.06)	**< 0.001**	**< 0.001**
Log_2_ humidity (%)	1.00 (0.99; 1.01)	0.241	0.281
2 weeks			
Log_2_ temperature (°C)	1.04 (1.03; 1.06)	**< 0.001**	**< 0.001**
Log_2_ humidity (%)	0.99 (0.98; 0.99)	**< 0.001**	**< 0.001**

Statistics: Association analyses were performed by conditional logistic regression analysis. *P*-values were corrected for multiple testing (*q*-values) using the false discovery rate (FDR) with Benjamini and Hochberg procedure

Abbreviations: 95% CI, 95% confidence interval; OR, odds ratio

**Fig. 2:**
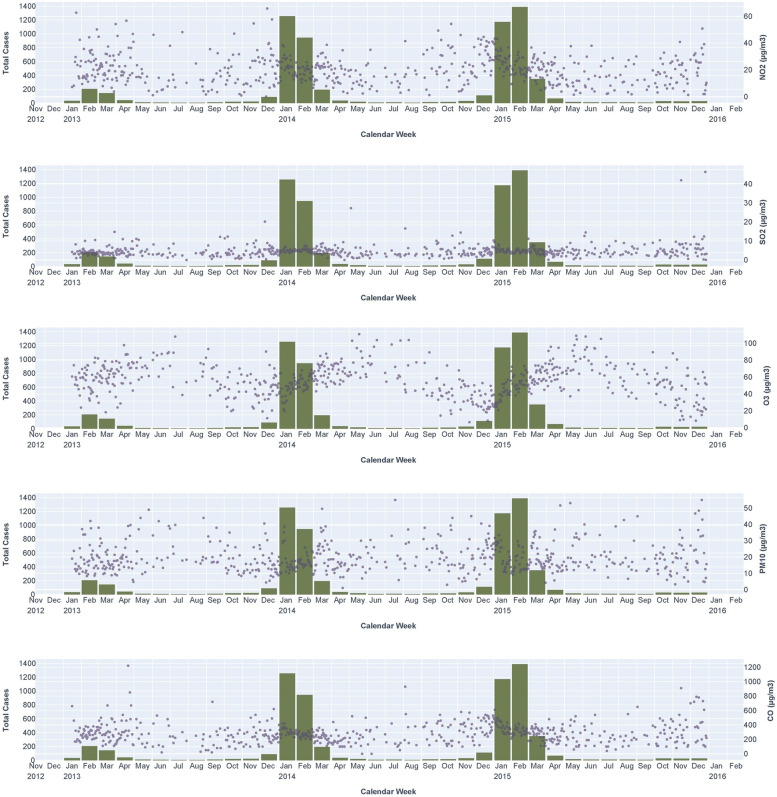
Summary of LRTI hospital admissions by months (green bars) and levels of ambient air pollutants (NO_2_, SO_2_, O_3_, PM_10_, and CO) by day (gray dots) in older people > 65 years from 2013 to 2015

**Fig. 3:**
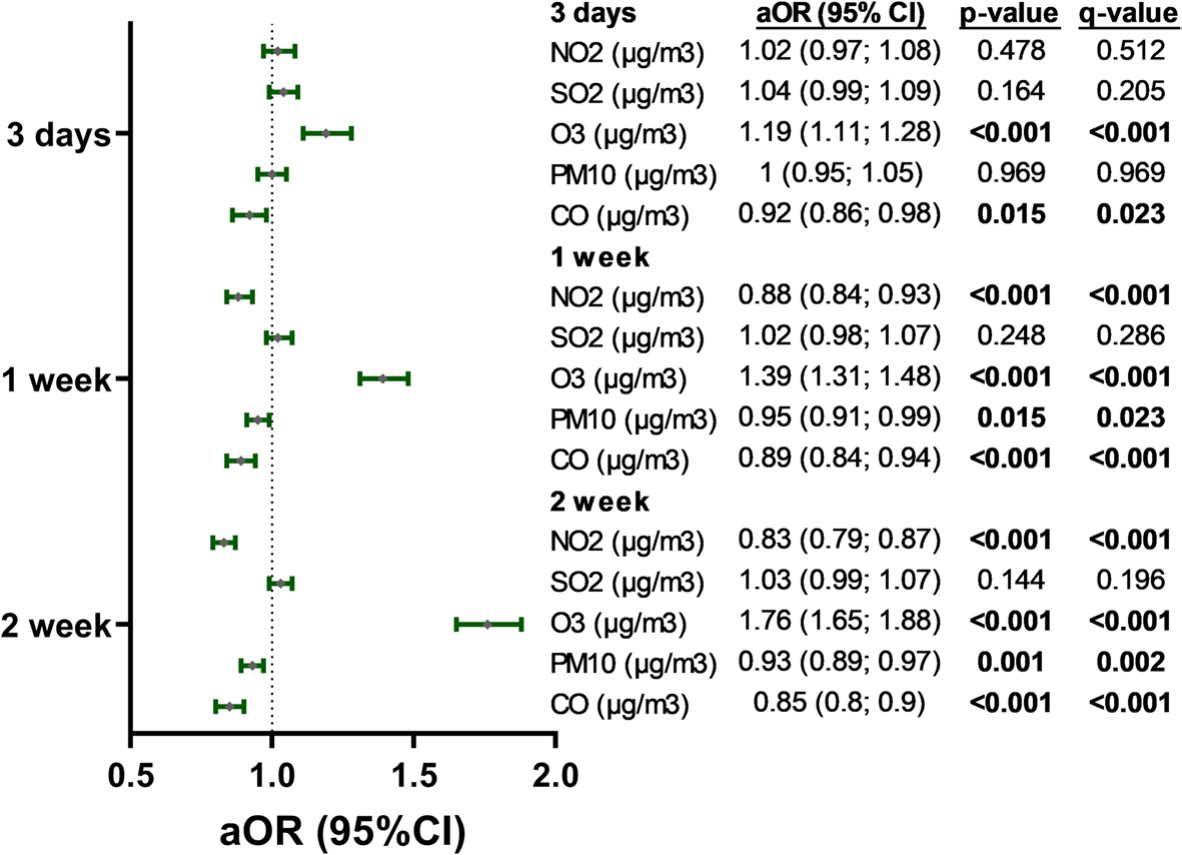
Relationship between environmental features and hospital admissions due to lower respiratory tract viral infections. Statistic: Multivariable conditional logistic regression was performed in three control lag times (3-days, 1-week, and 2-weeks before hospital admission). Relative humidity (%)], SO_2_ (μg/m^3^), CO (μg/m^3^), NO_2_ (μg/m^3^), O_3_ (μg/m^3^), and PM_10_ (μg/m^3^) were log_2_-transformed. *P*-values were corrected for false discovery rate (*q*-values). Abbreviations: 95% CI, 95% of the confidence interval; aOR, adjusted odds ratio

We also clearly observed that more LRTI hospital admissions were found when there were higher levels of NO_2_ and lower levels of O_3_. At the same time, SO_2_, PM_10_, and CO did not show a clear relationship between pollutants and LRTI hospital admissions (Fig. 2). The univariate and adjusted regression analysis between ambient air pollutants and LRTI hospital admissions showed similar OR values (Supplementary Table 2). The regression analysis adjusted by temperatures and relative humidity showed higher concentrations at the hospital admission for NO_2_ [compared to the lag time of 1-week (*q*-value< 0.001) and 2-weeks (*q*-value< 0.001)] and O_3_ [compared to the lag time of 3-days (*q*-value< 0.001), 1-week (*q*-value< 0.001), and 2-weeks (*q*-value< 0.001)] were related to a higher odds of hospital admissions due to viral LRTI (Fig. 3). Moreover, higher concentrations of PM_10_ at the lag time of 1-week (*q*-value = 0.023) and 2-weeks (*q*-value = 0.002), and CO at the lag time of 3-days (*q*-value = 0.023), 1-week (*q*-value< 0.001) and 2-weeks (*q*-value< 0.001)], compared to the day of hospitalization, were related to a higher chances of hospital admissions with viral LRTI (Fig. 3).

## Discussion

This study indicates that low temperatures, high relative humidity, and high concentrations of NO_2_, O_3_, PM_10_, and CO are associated with increased hospital admissions due to viral LRTI in patients 65 or older. Our data support the monitoring of environmental factors to assess the risk of hospital admissions and advise minimizing exposure to air pollutants in older people.

This study was performed for all 12 months, instead of only the colder months (December–March), when there were more hospitalized patients than during the warmer months (April–November). It is so because we wanted to analyze if there were associations between outdoor environmental pollution and LRTI hospitalizations at any time of the year (cold and warm seasons). As we showed, the epidemiological wave of viral LRTI occurs during the cold months (December–March), but there were also LRTI viral infections in the other months of the year, including summer.

Changes in weather conditions affect the respiratory system enabling the spread of infection-causing pathogens [7, 11]. These changes can increase the risk of viral LRTI and cause pneumonia, bronchitis, and other respiratory tract pathologies in older adults [9, 10]. An increase in the number of inflammatory cells and fibrinogen concentration has been observed during cold exposure, damaging the respiratory system, and leading to urgent hospitalizations and possible death [7, 11]. Besides, lower temperatures increase pathogens’ stability, abundance, survival, and infectivity [7]. High humidity increases the infectivity of viruses because humidity stabilizes the droplets that carry the pathogen from person to person through the air [7]. Our study found a higher risk of hospitalization for viral LRTI among older adults ≥65 years exposed to low temperatures and high relative humidity before hospital admission. Low temperatures and high humidity are associated with a higher risk of viral LRTI [24–26]. Our data agree with previous data showing that ambient temperatures below the reference levels potentiate respiratory tract infections and increase hospital admissions in older adults [7, 9, 10]. However, some studies show discordant data on temperature concerning our research [27, 28], partially justified because not all regions of the world have the same seasonal pattern of LRTI, finding differences in the circulation of respiratory viruses according to geographic characteristics [29–31].

NO_2_ is an irritating pollutant related to the high traffic that penetrates deep into the lung, causing respiratory diseases, including viral LRTI [2, 6]. NO_2_ causes an imbalance in the Th1/Th2 differentiation (increased IL-4/IFN-γ ratio) and the activation of the JAK-STAT pathway, damaging the lung cell membrane and increasing airway inflammation [32]. Our study found an elevated risk of hospital admissions due to viral LRTI associated with short-term exposure to NO_2_ in older people. Our findings are consistent with other reports on short-term [33] and long-term [34] exposure to outdoor NO_2_ and COVID-19 in older people with respiratory failure. It may be due to NO_2_ inhalation oxidizing proteins and lipids and altering the immune system [35]. However, discordant studies did not show any association between outdoor NO_2_ and LRTI in older people [36], suggesting that outdoor NO_2_ may impact viral LRTI in combination with other environmental pollutants rather than NO_2_ itself [37, 38].

O_3_ is a potent and toxic oxidizing gas that arises in the stratosphere or the troposphere after various reactions from photochemical smog [2, 6]. Its absorption usually occurs by inhalation, which can penetrate deeply into the lungs due to its low solubility in water. O_3_ reacts with cells lining the airways, stimulating their receptors and nerve endings and leading to oxidative stress, inflammation, and decreased total lung capacity [39]. Our findings are consistent with previous reports that found significant associations between short-term exposure to ambient O_3_ and increased risk of pneumonia hospital admissions among older adults [40, 41]. However, discordant studies did not find a relationship between outdoor O_3_ and LRTI hospital admissions [42, 43].

In our study, O_3_ was the most critical environmental factor because it was strongly associated with viral LRTI hospital admissions, increasing with longer delay times. Interestingly, the epidemiological wave of viral LRTI occurred during the cold months (December–March), when O_3_ levels were lower compared to the warmer and hotter months (May–September) when older people spend much more time outdoors. The impact of O_3_ on the LRTI severity depends on several factors, such as viral epidemiological characteristics and O_3_ exposure (outdoor activities, O_3_ concentrations, exposure time, and susceptibility to air pollutants). The O_3_ sources in winter are practically the same as in summer, mainly for chemical reactions between O_3_ precursors in the atmosphere, such as NO_X_ and volatile organic compounds from combustion associated with cars, planes, trains, power plants, oil refineries, factories, or evaporation of organic compounds from standard consumer products (paints, cleaning products, and solvents) [44]. O_3_ levels increase when their precursor emissions react in the presence of sunlight, warm temperatures, and light winds (warm seasons). When winter arrives, the temperature and solar radiation decrease, and most of the warm air rise, displacing O_3_ to the upper layers of the atmosphere [44]. However, it should also be noted that Spain has a Mediterranean climate characterized by hot summers, low winds, and intense solar radiation; and cool winters that are slightly cloudy and rainy. It affects the physical-chemical processes of O_3_ formation, which is why O_3_ continues to be generated in the cold months, with production peaks on specific days when the temperature and solar radiation are higher [45].

PM_10_ can be inhaled through small liquid or solid droplets that invade the lungs and cause long-term severe respiratory problems. PM_10_ has a long half-life, allowing it to spread to distant destinations, where people become exposed [2, 6]. PM_10_ causes lung damage by increasing inflammation and airspace epithelial permeability [46]. Several studies have demonstrated an association between particulate matter up to 2.5 μm in size (PM_2.5_) and emergency visits for severe viral respiratory diseases in older patients [34, 47, 48]. Unlike our study, other studies reported no increase in LRTI hospitalizations related to PM_10_ [8], likely due to varying ambient PM_10_, weather conditions, and co-pollutants in different geographic areas.

CO is generated mainly during incomplete hydrocarbon combustion from internal combustion engines, waste incinerators, coal power plants, and the oil industry. CO diffuses quickly across the pulmonary membrane triggering proinflammatory responses in the airways [49]. CO is a “silent killer” that binds to hemoglobin in the blood, forming carboxyhemoglobin that displaces oxygen, reduces oxygen-carrying capacity, and decreases the release of oxygen to tissues, increasing the risk of asphyxia-related deaths [50]. Inhalation of CO can be toxic to the respiratory system, causing asthma exacerbation [51] and increased hospital admission for chronic obstructive pulmonary disease [52]. Our data concord with other studies that found an association between outdoor CO levels and hospital admissions for viral LRTI [53–55] and pneumonia [56]. Nevertheless, another report has not shown significant associations between CO and respiratory and LRTI hospital admissions [57–59]. These controversial results can be due to densely populated areas, urban congestion, and heavy traffic load, where the predominant air pollutants are NO2 and particulate matter. Therefore, the effects of the CO’s co-emission with these airborne pollutants may confound the contribution of CO in air pollution on health [60].

### Strengths and limitations of the study

Our study also has several strengths that must be considered: (i) this is a nationwide study with a very high number of older adults over 65 years of age with a viral LRTI hospital admission, something challenging to reach with any other database; (ii) we use a bidirectional case-crossover design that minimizes the impact of the absence of fundamental variables in the regression analysis [21].

The most important limitations are the following: (i) The retrospective design may introduce biases; (ii) the lack of relevant clinical information for the correct interpretation of the data since medical history data (comorbidities and treatments) may affect hospital admission and a stratified analysis would have provided exciting information in this regard; (iii) the diagnostic bias because in the MBDS there was no specific code for the diagnosis of LRTI, and we used ICD-9-CM codes previously used in high impact factor publications [47, 61], but we do not really know the accuracy of the MBDS for LRTI diagnoses; (iv) the MBDS is anonymous and makes it difficult to control whether some older people over 65 have been hospitalized several times; (v) we did not analyze other emerging outdoor air pollutants, such as volatile organic compounds, including benzene; and (vi) lack of indoor air pollution data may have a significant impact on viral LRTIs because most people, especially the older population, spent more time indoors [62], facilitating the transmission of viral LRTIs among everyone.

## Conclusions

Unfavorable environmental factors (low temperatures, high relative humidity, and high concentrations of NO_2_, O_3_, PM_10_, and CO) increased the odds of hospital admissions with viral LRTI among older people, indicating they are potentially vulnerable to these environmental factors.

## Supplementary Information

**Additional file 1:** **Supplementary Table 1.** Summary of ICD-9-CM coding used for baseline comorbidities investigated in this study.

**Additional file 2:** **Supplementary Table 2.** Summary of adjusted associations between ambient air pollutants and hospital admissions for lower respiratory tract viral infections in Spain (2013–2015).

## Data Availability

All relevant data is contained in the article and supplemental files. Those interested can contact Dr. Alejandro Alvaro Meca for additional information at alejandro.alvaro@urjc.es. The MDBS is the property of the MHCSW and the environmental data of the AEMET. Therefore, any researcher can request the data related to this article from MHCSW and AEMET.

## Abbreviations

95%CI: 95% Confidence interval
AEMET: State Meteorological Agency
CO: Carbon monoxide
COVID-19: Coronavirus Disease 2019
Flu: Influenza virus
ICD-9-CM: International Classification of Diseases, 9th ed., Clinical Modification
ICU: Intensive care unit
IFN-γ: Interferon gamma
IL-4: Interleukin 4
JAK-STAT: Janus kinase-signal transducer and activator of transcription
LRTI: Lower respiratory tract infection
MBDS: Minimum Basic Data Set
MHCSW: Ministry of Health, Consumer Affairs, and Social Welfare
NO_2_: Nitrogen dioxide
O_3_: Ozone
OR: Odds ratio
PM_10_: Particulate matter up to 10 μm in size
PM_2.5_: Particulate matter up to 2.5 μm in size
RNS: Reactive nitrogen species
ROS: Reactive oxygen species
RSV: Respiratory syncytial virus
SO_2_: Sulfur dioxide
Th1/Th2: T helper cell type 1/type 2
