# Supplementary material for: Neighborhood environmental factors linked to hospitalizations of older people for viral lower respiratory tract infections in Spain: a case-crossover study
Source: Environ Health. 2022 Nov 8;21:107. doi: 10.1186/s12940-022-00928-x (PMC9640778; doi:10.1186/s12940-022-00928-x)
Supplement: Supplementary file 1 — Additional file 1: Supplementary Table 1. Summary of ICD-9-CM coding used for baseline comorbidities investigated in this study. [file 12940_2022_928_MOESM1_ESM.docx]

**Supplementary Table 1**. Summary of ICD-9-CM coding used for baseline comorbidities investigated in this study.

| **Description** | **Diagnosis codes (index or prior admissions)** |
| --- | --- |
| **Charlson comorbidities** |  |
| Myocardial infarction | 410.x, 412.x |
| Congestive heart failure | 428.x |
| Peripheral vascular disease | 443.9, 441.x, 785.4, V43.4, Procedure 38.48 |
| Cerebrovascular disease | 430.x–438.x |
| Dementia | 290.x |
| Chronic pulmonary disease | 490.x–505.x, 506.4 |
| Rheumatic disease | 710.0, 710.1, 710.4, 714.0–714.2, 714.81, 725.x |
| Peptic ulcer disease | 531.x–534.x |
| Mild liver disease | 571.2, 571.4–571.6 |
| Diabetes without chronic complication | 250.0–250.3, 250.7 |
| Diabetes with chronic complication | 250.4–250.6 |
| Hemiplegia or paraplegia | 344.1, 342.x |
| Renal disease | 582.x, 583–583.7, 585.x, 586.x, 588.x |
| Any malignancy, including lymphoma and leukemia, except malignant neoplasm of skin | 140.x–172.x, 174.x.–195.8, 200.x–208.x |
| Moderate or severe liver disease | 456.0–456.21, 572.2–572.8 |
| Metastatic solid tumor | 196.x–199.1 |
| AIDS/HIV | 042.x–044.x |
| **Lower respiratory tract viral infections** |  |
| Respiratory syncytial virus | 079.6, 466.11, 480.1 |
| Influenza | 487.0, 487.1, 488.01, 488.02, 488.11, 488.12, 488.81, 488.82 |
| Viral pneumonia | 480.0, 480.1, 480.2, 480.8, 480.9 |
| Acute bronchiolitis | 466.11 466.19 |
| Acute respiratory failure | 518.81 |
