# Supplementary material for: Neighborhood environmental factors linked to hospitalizations of older people for viral lower respiratory tract infections in Spain: a case-crossover study
Source: Environ Health. 2022 Nov 8;21:107. doi: 10.1186/s12940-022-00928-x (PMC9640778; doi:10.1186/s12940-022-00928-x)
Supplement: Supplementary file 2 — Additional file 2: Supplementary Table 2. Summary of adjusted associations between ambient air pollutants and hospital admissions for lower respiratory tract viral infections in Spain (2013–2015). [file 12940_2022_928_MOESM2_ESM.docx]

**Supplementary Table 2.** Summary of adjusted associations between ambient air pollutants and hospital admissions for lower respiratory tract viral infections in Spain (2013-2015).

|  | **Univariate** | | | **Multivariate** | | |
| --- | --- | --- | --- | --- | --- | --- |
| **3 days** | **OR (95% CI)** | ***p*-value** | ***q*-value** | **aOR (95% CI)** | ***p*-value** | ***q*-value** |
| Log_2_ NO_2_ (μg/m^3^) | 1.01 (0.96; 1.07) | 0.635 | 0.702 | 1.02 (0.97; 1.08) | 0.478 | 0.512 |
| Log_2_ SO_2_ (μg/m^3^) | 1.04 (0.98; 1.09) | 0.173 | 0.227 | 1.04 (0.99; 1.09) | 0.164 | 0.205 |
| Log_2_ O_3_ (μg/m^3^) | 1.18 (1.10; 1.27) | **<0.001** | **<0.001** | 1.19 (1.11; 1.28) | **<0.001** | **<0.001** |
| Log_2_ PM_10_ (μg/m^3^) | 0.99 (0.95; 1.05) | 0.996 | 0.996 | 1 (0.95; 1.05) | 0.969 | 0.969 |
| Log_2_ CO (μg/m^3^) | 0.91 (0.85; 0.98) | **0.009** | **0.015** | 0.92 (0.86; 0.98) | **0.015** | **0.023** |
| **1 week** |  |  |  |  |  |  |
| Log_2_ NO_2_ (μg/m^3^) | 0.88 (0.83; 0.92) | **<0.001** | **<0.001** | 0.88 (0.84; 0.93) | **<0.001** | **<0.001** |
| Log_2_ SO_2_ (μg/m^3^) | 1.03 (0.99; 1.07) | 0.196 | 0.242 | 1.02 (0.98; 1.07) | 0.248 | 0.286 |
| Log_2_ O_3_ (μg/m^3^) | 1.36 (1.28; 1.45) | **<0.001** | **<0.001** | 1.39 (1.31; 1.48) | **<0.001** | **<0.001** |
| Log_2_ PM_10_ (μg/m^3^) | 0.96 (0.91; 0.99) | **0.039** | 0.059 | 0.95 (0.91; 0.99) | **0.015** | **0.023** |
| Log_2_ CO (μg/m^3^) | 0.88 (0.83; 0.93) | **<0.001** | **<0.001** | 0.89 (0.84; 0.94) | **<0.001** | **<0.001** |
| **2 weeks** |  |  |  |  |  |  |
| Log_2_ NO_2_ (μg/m^3^) | 0.82 (0.78; 0.86) | **<0.001** | **<0.001** | 0.83 (0.79; 0.87) | **<0.001** | **<0.001** |
| Log_2_ SO_2_ (μg/m^3^) | 1.03 (0.99; 1.07) | 0.151 | 0.211 | 1.03 (0.99; 1.07) | 0.144 | 0.196 |
| Log_2_ O_3_ (μg/m^3^) | 1.74 (1.63; 1.85) | **<0.001** | **<0.001** | 1.76 (1.65; 1.88) | **<0.001** | **<0.001** |
| Log_2_ PM_10_ (μg/m^3^) | 0.93 (0.89; 0.97) | **0.001** | **0.002** | 0.93 (0.89; 0.97) | **0.001** | **0.002** |
| Log_2_ CO (μg/m^3^) | 0.83 (0.79; 0.88) | **<0.001** | **<0.001** | 0.85 (0.8; 0.9) | **<0.001** | **<0.001** |

**Statistics:** Association analyses were performed by conditional logistic regression analysis. *P*-values were corrected for multiple testing (*q*-values) using the false discovery rate (FDR) with Benjamini and Hochberg procedure.

**Abbreviations:** 95% CI, 95% confidence interval; aOR, adjusted odds ratio.
